# Percutaneous nephrostomy for complex renal stones: Percutaneous renal access behind the stone versus renal calyx dilation

**DOI:** 10.1371/journal.pone.0278485

**Published:** 2022-12-01

**Authors:** Jae Kyeong Ahn, Jung Ho Won, Dae Seob Choi, Ho Cheol Choi, Hye Young Choi, Sa Hong Jo, Jae Hwi Choi, Seung Hye Lee, Mi Ji Kim, Sung Eun Park, Ji Hoon Shin

**Affiliations:** 1 Department of Radiology, Gyeongsang National University School of Medicine and Gyeongsang National University Hospital, Jinju, Korea; 2 Department of Urology, Gyeongsang National University School of Medicine and Gyeongsang National University Hospital, Jinju, Korea; 3 Department of Internal Medicine-Nephrology, Gyeongsang National University School of Medicine and Gyeongsang National University Hospital, Jinju, Korea; 4 Department of Preventive Medicine, Institute of Health Sciences, Gyeongsang National University School of Medicine, Jinju, Korea; 5 Department of Radiology, Gyeongsang National University School of Medicine and Gyeongsang National University Changwon Hospital, Changwon, Korea; 6 Department of Radiology and Research Institute of Radiology, Asan Medical Center, University of Ulsan College of Medicine, Seoul, Korea; Aurora Medical Center Bay Area, UNITED STATES

## Abstract

**Objective:**

To evaluate the technical success rate and complications associated with percutaneous nephrostomy (PCN) via percutaneous renal access behind the stone and renal calyx dilation in patients with complex renal stones.

**Materials and methods:**

From January 2010 to February 2021, we identified 69 patients with 70 complex renal stones who underwent PCN. Complex renal stones were classified as simple (renal pelvis only) (27.1%, 19/70), borderline staghorn (8.6%, 6/70), partial staghorn (51.4%, 36/70), or complete staghorn (12.9%, 9/70). All PCNs were performed under ultrasound and fluoroscopic guidance using one of two renal-entry techniques: puncture behind the stone (56%, 39/70) or renal calyx dilation (44%, 31/70). Then, we retrospectively evaluated the technical success rates and complications associated with each renal entry access technique.

**Results:**

The overall technical success rate was 100%, and the complication rate was 20.0% (14/70). For those who underwent renal access behind the stone, the complication rate was 15.4% (6/39), and six patients (six PCNs) had transient gross hematuria. For those who underwent dilated renal calyx entry, the complication rate was 25.8% (8/31), and one patient had significant bleeding complications requiring transfusion. Furthermore, seven patients (seven PCNs) had transient gross hematuria. Overall, the complication rates did not differ between the technique groups (p = 0.279)

**Conclusion:**

PCN for complex renal stones has a high technical success rate and an acceptable complication rate regardless of the specific technique. Renal entry behind the stone is as safe and feasible as approaching via a dilated renal calyx.

## Introduction

Complex renal stones, including staghorn stones, are large and branched stones that occupy all or part of the renal pelvis and calyces [1, 2]. Furthermore, they can be asymptomatic and induce urinary tract infections, urinary tract obstruction, hematuria, and decreased kidney function [2, 3]. If left untreated or conservatively treated, complex renal stones can lead to an increased risk of renal failure and mortality [2–5]. Therefore, complex renal stones require aggressive treatment [2–5].

Many surgical and non-surgical treatment options have been introduced to remove complex renal stones, including percutaneous nephrolithotomy (PCNL), shock wave lithotripsy (SWL), PCNL and SWL combinations, and open surgery [1, 2]. PCNL is the first-choice treatment for large and complex renal stones [1, 4, 6, 7], but percutaneous nephrostomy (PCN), a well-established renal access technique, must precede PCNL [8]. PCN is relatively safe and easy for a dilated renal collecting system [9]. However, few reports have described PCN for complex renal stones [10, 11]. Moreover, those few reports emphasized the difficulty of PCN for complex renal stones, reporting a technical success rate of 82 to 85% compared with 96 to 100% for a dilated renal system [10, 11].

Ultrasound imaging is a well-established renal pathology evaluation method [12].

Specifically, ultrasound imaging reliably and efficiently detects larger renal stones exhibiting posterior acoustic shadowing [12]. Therefore, percutaneous renal access behind a stone allows efficient renal entry under ultrasound guidance [13].

Therefore, to clarify the efficacy and safety of PCN with complex renal stones, we compared the technical success rate and complications associated with two rental entry approaches: renal entry behind the stone and renal calyx dilation.

## Materials and methods

### Subjects

The Institutional Review Board of the University of Gyeongsang National University Hospital approved this retrospective study and waived the requirement for informed consent since we used only de-identified data collected during clinical practice (IRB No. GNUH 2022-06-009).

### Complex renal stone classifications

Generally, complex renal stones are calculi greater than 3.0 cm in diameter with multiple renal stones, staghorn stones, abnormally positioned renal calculi, or horseshoe kidney calculi in a single kidney [14–16]. Staghorn stones are the most complex renal stones, defined as branch stones of the renal collecting system and subclassified as borderline, partial, or complete staghorn stones [5, 15]. Borderline staghorn stones were defined as those filling the pelvis and one calyx. Partial staghorn stones were defined as stones filling at least two branched calyces and 40% of the renal collecting system. Finally, a complete staghorn stone was defined as a stone filling all major calyces in at least 80% of the renal collecting system [3, 5, 15]. Therefore, we classified the complex renal stones as renal pelvis only, borderline staghorn, partial staghorn, and complete staghorn.

### Patient selection

Between January 2010 and February 2021, 1371 PCNs were performed during this period. Finally we identified 69 patients who underwent percutaneous nephrostomy for complex renal stones. One patient had complex, bilateral renal stones. The selected patients met the criteria for renal calculi greater than 3.0cm in diameter. The exclusion criteria as follows: 1. PCNs were performed for other reasons such as malignant urinary obstruction (564 PCNs), urinary tract infection (125 PCNs), ureteral stricture (55 PCNs) and others (94 PCNs: ureteral injury, neurogenic bladder, double J stent malfunction, etc.) 2. PCNs were performed for renal calculi smaller than 3.0cm in diameter (463 PCNs).

All patients underwent routine computed tomography or kidney, ureter, and bladder imaging to evaluate the anatomy of the kidney, the locations of the stones, and the positions of the adjacent structures in relation to the desired puncture route. None of the patients had severe coagulopathy (international normalized ratio, >1.5; platelet count, <50,000/mm^3^ or took oral anticoagulants.

### The PCN placement technique

All PCN procedures were performed by one of three experienced interventional radiologists with 4–16 years of clinical experience. A percutaneous approach through the posterior calyx in the lower or middle pole was preferred in all cases to prevent vascular injury [13]. All PCN procedures were performed under ultrasound and fluoroscopic guidance.

**Fig 1:**
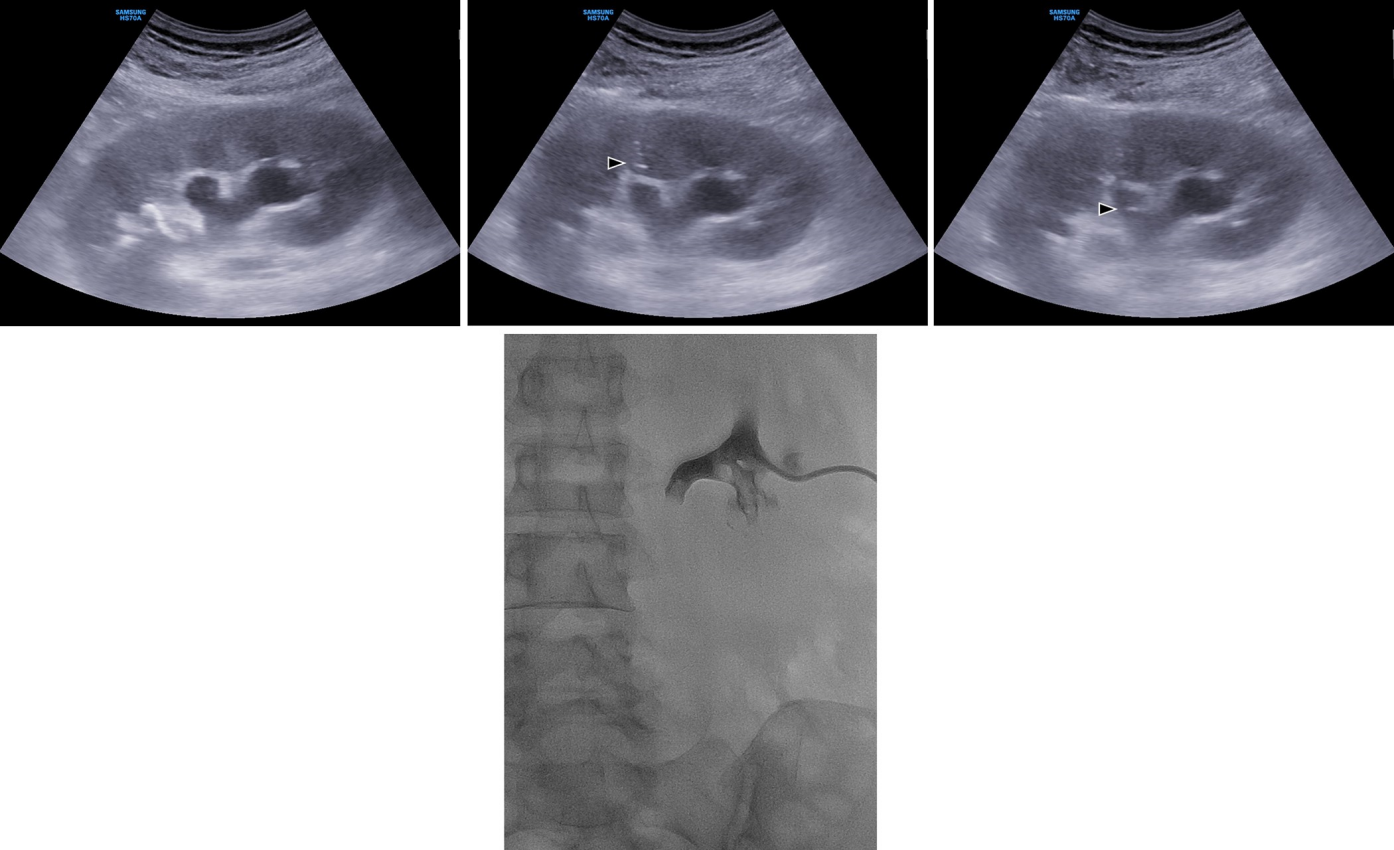
Renal access for dilated renal calyx with hydronephrosis under ultrasound guidance. (A) A grayscale ultrasonography image showed a dilated middle and superior renal calyces and replaced inferior renal calyx with renal stone in the right kidney. (B) A 21-G Chiba needle (arrowhead) was advanced to the center of the renal pyramid at middle pole. (C)The Chiba needle (arrowhead) was introduced into the dilated renal calyx. (D) Finally an 8.5Fr drainage catheter was successfully inserted after renal access via dilated renal calyx.

Renal access was performed under ultrasound guidance using a 21G Chiba needle (Cook Medical Inc., Bloomington, IN, USA) to puncture the renal collecting system. The procedures after Chiba needle insertion were performed under fluoroscopic guidance. For dilated renal calyces with hydronephrosis, renal access was the same as PCN for a dilated renal collecting system, and the Chiba needle targeted the dilated renal calyces (Fig 1) [17]. However, if the renal calyces were replaced with complex stones with little hydronephrosis, the Chiba needle targeted the center of the renal pyramid behind the stone.

**Fig 2:**
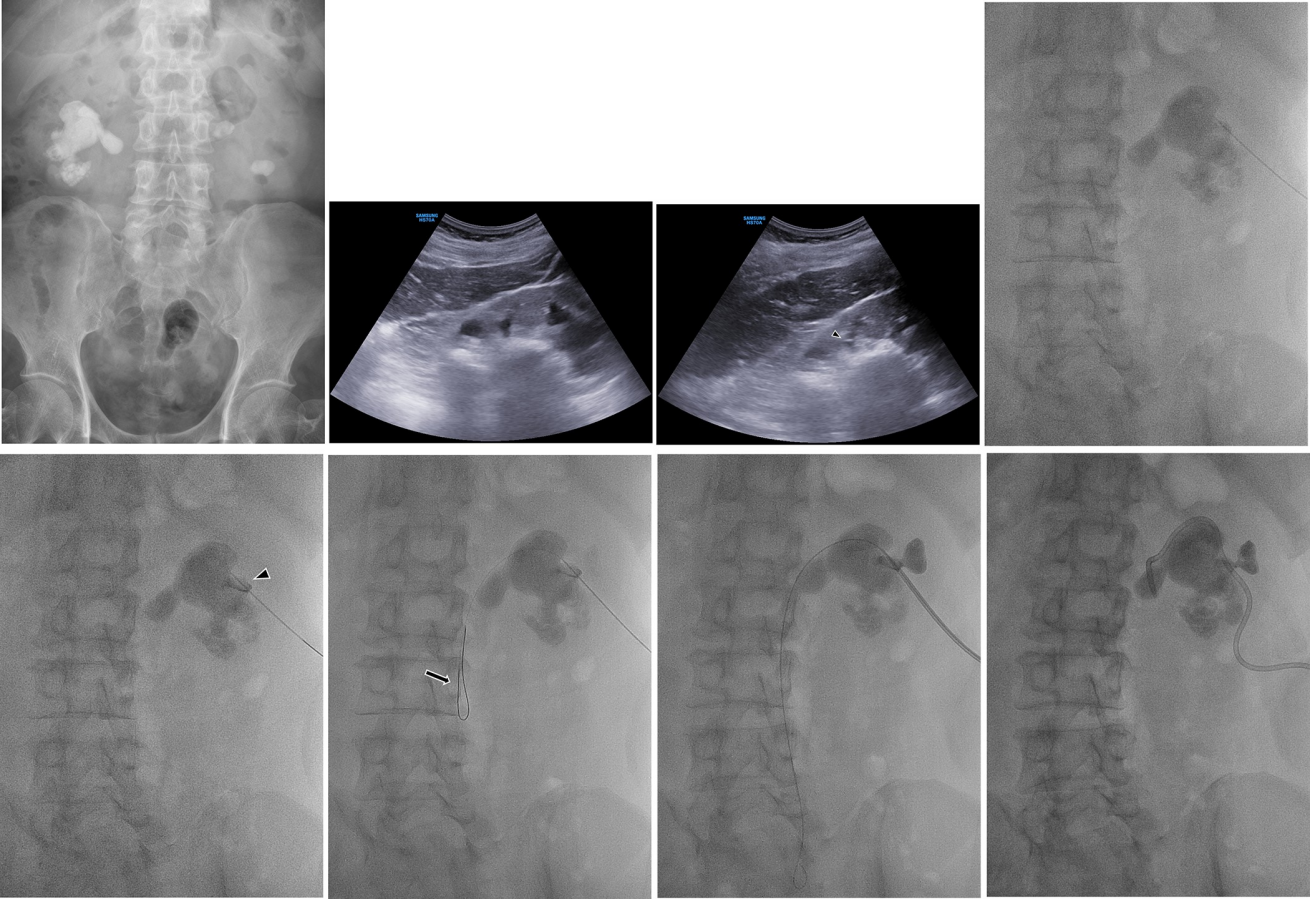
A representative case of a 61-year-old man with a right, partial staghorn stone. (A) A kidney, ureter, and bladder imaging exam identified a right, partial staghorn stone. (B) A grayscale ultrasonography image showed a staghorn stone in the right kidney with marked posterior acoustic shadowing. (C) A 21-G Chiba needle (arrowhead) was introduced into the periphery of the complex stone through the center line of the renal pyramid. (D) A radiograph image showed that the 21-G Chiba needle was introduced behind the stone. (E) A few millilters of contrast medium was injected, then the renal calyx was opacified by contrast medium (arrowhead). (F) A 0.018-inch guide wire (arrow) was introduced with rotating movements to minimize resistance at the tip as the wire advanced. (G) A 0.035-inch hydrophilic guidewire was introduced, and track dilatation was performed under fluoroscopic guidance. (H) An 8.5-Fr pigtail catheter was placed after sequential tract dilatation.

All PCNs were performed with the patient in the prone position. First, ultrasound scanning was performed using a 7.5 MHz transducer to obtain a signal through the kidney. Once the initial puncture site was selected, it was cleaned and draped. Then, local anesthesia with 2% lidocaine was injected at the puncture site. When renal calyces were observed on ultrasound, a 21-G Chiba needle was advanced to the dilated renal calyx (i.e., the rental entry via renal calyx dilation group). If complex renal stones with little hydronephrosis replaced renal calyces (Fig 2B), then a 21-G Chiba needle was introduced into the periphery of the complex renal stone through the center line of the renal pyramid (i.e., the renal entry behind the stone group; Fig 2C and 2D). The urine from hydronephrosis was drained after the inner stylet was removed. If the Chiba needle did not induce a release of urine due to complex stones with little hydronephrosis, a few milliliters of contrast medium was injected to ensure proper positioning of the needle tip and opacify the targeted renal calyx under fluoroscopic guidance (Fig 2E). Once the position of the needle was ensured, a 0.018-inch guidewire was introduced through the needle with rotating movement to minimize resistance at the tip as the wire advanced to accommodate the complex renal stone (Fig 2F). Next, the needle was exchanged with a 5Fr introducer sheath (A&A Medical Device Company, Seongnam, Korea), followed by a 0.035-inch hydrophilic guide wire (Terumo Corporation, Tokyo, Japan) into the renal pelvis or proximal ureter. Once the guidewire was in position, an 8Fr dilator (Cook Medical Inc.) was inserted for tract dilatation (Fig 2G). After tract dilation, an 8.5Fr pigtail catheter (Cook Medical Inc.) was introduced over the guidewire (Fig 2H). All procedures were completed by confirming that the PCN catheter tip was in the renal pelvis near the proximal ureter on the final radiograph. A successful PCN was defined as satisfactory placement of a PCN catheter in the renal pelvis with successful opacification of the renal collecting system on antegrade pyelography.

### Data collection

We collected data on the technical success rates and associated complications. Bilateral PCNs for patients with two complex renal stones were considered two different PCNs. Technical successes and the number of complications were counted and evaluated as percentages. Complications were classified based on the Society of Interventional Radiology (SIR) guidelines [11]. Minor complications were complications that did not require therapy and were without further consequences or required nominal therapy, also without further consequences (e.g., overnight admission for observation only). Major complications were complications requiring therapy with minor hospitalization (<48 hours), major therapy with an unplanned increase in care and prolonged hospitalization (>48 hours), and complications resulting in permanent adverse sequelae or death.

The follow-up period was the time from the primary catheter until PCNL, another method for complex renal stone removal, or the first hospitalization for PCN.

Statistical analyses were performed using a one-sample proportion test and Pearson’s chi-square test for categorical and continuous data, respectively. A P-value of <0.05 was considered statistically significant.

## Results

Overall, 39 patients underwent PCN via puncture behind the stone, and 31 underwent PCN via puncture of the dilated renal calyx. Table 1 presents the baseline demographics. Overall, the technical success rate was 100%. During the follow-up period (mean, 12 days; range 1–58 days), 40 patients (40 PCNs) underwent PCNL, and 14 patients (14 PCNs) underwent other surgeries for stone removal, including ureterolithotomy or extracorporeal shockwave lithotripsy. Furthermore, 15 patients (16 PCNs) underwent only conservative treatment because of older age and accompanying malignancies. One patient died four days after PCN due to old age, pneumonia, and acidosis progression.

**Table 1:** Patient demographics.

Characteristics	PCN via behind the stone n (%)	PCN via dilated renal calyx n (%)
Patients (n)	38	31
Age (years)	66.7 ± 13.2	68.3 ± 14.3
**Gender**		
Male	22 (57.9)	15 (48.4)
Female	16 (42.1)	16 (51.6)
**Numbers of kidneys**		
Left	21 (55.3)	15 (48.4)
Right	16 (42.1)	16 (51.6)
Both	1 (2.6)	
**Renal stone classification**		
Renal pelvis only	0	19 (61.3)
Borderline staghorn	1 (2.6)	5 (16.1)
Partial staghorn	29 (74.3)	7 (22.6)
Complete staghorn	9 (23.1)	0
**Renal entry selection**		
Upper pole	0	0
Mid pole	7 (18.0)	10 (32.3)
Lower pole	32 (82.0)	21 (67.7)

Abbreviations: PCN, percutaneous nephrostomy

Table 2 presents the complication rates per group. The overall complication rate was 20.0% (14/70). Furthermore, the complication rate was 15.4% (6/39) for the group with renal access behind the stone; none had major complications, and 6 patients had minor complications. However, 6 patients (6 PCNs) had transient gross hematuria. The complication rate was 25.8% (8/31) for the dilated renal calyx group; 1 patient had major bleeding complications that required transfusion. After the procedure, the patient’s hemoglobin level decreased from 9.6 g/dL to 6.9 g/dL. Therefore, one unit of red blood cells was transfused. The hemoglobin level recovered to 8.4 g/dL, and the patient stabilized. Furthermore, 7 patients had transient gross hematuria. Overall, there was no mortality, and all transient gross hematuria resolved within 72 hours. Finally, the complication rates did not differ between the two percutaneous access techniques (p = 0.279).

**Table 2:** PCN complications based on the renal approach technique.

	Behind the stone (n = 39)	Dilated renal calyx (n = 31)	P-value
Total complications (%)	6 (15.4)	8 (25.8)	0.279
**Major complications**			
Bleeding requiring transfusion	0	1 (3.2)	
**Minor complications**			
Transient gross hematuria	6 (15.4)	7 (22.6)	

Abbreviations: PCN, percutaneous nephrostomy

## Discussion

PCNL is the preferred option for complex renal stones or stones resistant to SWL or ureteroscopy [1, 4, 6, 7, 18]. Proper access to the renal collecting system plays a crucial role in the procedure’s success [18]. However, little attention has been paid to percutaneous renal access. Thus, we compared two percutaneous renal access techniques: entry behind the stone and entry via a dilated renal calyx, which, to our knowledge, has not been done before.

In this study, the percutaneous nephrostomy technical success rate for complex renal stones under ultrasound and fluoroscopic guidance was 100%, higher than the 82 to 85% technical success rate previously reported [11]. Furthermore, our success rate was higher than the 80% minimum threshold set by the SIR for PCN with complex stone disease and staghorn calculi [11]. This study used a simultaneous ultrasound and fluoroscopic guidance approach for percutaneous access. Traditionally, the puncture during PCNL is performed under fluoroscopic guidance [19, 20], but there are disadvantages to this approach, including radiation exposure and possible iatrogenic visceral injury to the colon, liver, spleen, and pleura [19–23]. Ultrasound-guided percutaneous access is an alternative method associated with less radiation exposure and possible injury to nearby organs [20]. However, a few studies reported difficulties in ultrasound-guided (USG) PCNL with a non-dilated collecting system [24, 25], concluding that USG PCNL is safe for patients with a single stone at the renal pelvis in moderately to markedly dilated renal calyces [25]. When renal access occurs via the underlying stone, the renal approach targets the dilated renal calyx. However, if the renal calyx is filled with a stone instead of urine, the calyceal USG findings differ. For example, we identified a hyperechoic stricture (stone) accompanied by posterior shadowing and a hypoechoic stricture filled with urine (hydronephrosis).

During the procedure, it is best to aim for the center of the renal pyramid behind the stone because the Chiba needle passing through a hyperechoic stone is almost invisible by USG imaging. Furthermore, the renal pyramid center projects onto a minor calyx [26]. Therefore, a puncture through the renal pyramid midline goes through the axis of the corresponding minor calyx obscured by the complex renal stone.

Table 2 details the reported complications. There were no major complications in the group that underwent renal access behind the stone. However, one patient experienced a major complication (3.2%) in the group that underwent access by dilated renal calyx, which was acceptable considering that the SIR recommends a major complication rate of less than 4% [11]. Moreover, transient gross hematuria resolved within 72 hours in all cases, which was considered a clinically minor complication since the hematuria improved with conservative treatment regardless of the technique. The minor complication rates were 15.4% for the behind-the-stone approach and 25.8% for the dilated renal calyx approach, higher than the 15% upper margin for minor complications recommended by the Royal College of Radiologists [27]. However, the rates correspond with the reported 5.3 to 28% incidence rate during general PCN procedures [28–32]. Finally, septic shock, vascular injuries requiring embolization, individual threshold complications resulting in an unexpected transfer to the intensive care unit, or emergency surgery related to the PCN procedure did not occur.

This study has several limitations. First, this study retrospectively reviewed the clinical and imaging findings. Therefore, data from some clinical charts and notes were limited and could not be included. Second, PCN placement was performed by three interventional radiologists with differing degrees of experience in PCN procedures for complex renal stones, although they had sufficient clinical experience. Third, data on radiation exposure during PCN were not fully obtained. Thus, we were limited to analyzing radiation exposure during each PCN procedure.

## Conclusion

PCN for complex renal stones has a high technical success rate and an acceptable complication rate regardless of the specific technique. Renal entry behind the stone is as safe and feasible as the dilated renal calyx approach.

## Supporting information

S1 Data (XLSX)
